# BTN2A2, a new biomarker and therapeutic target for glioma

**DOI:** 10.18632/aging.205039

**Published:** 2023-10-17

**Authors:** Heping Wang, Shanrui Pu, Haitao Xu, Lihong Yang, Lishi Shao, Xi Chen, Xiaobin Huang, Jun Pu

**Affiliations:** 1 The First Department of Neurosurgery, The Second Affiliated Hospital of Kunming Medical University, Kunming 650223, China; 2 NHC Key Laboratory of Drug Addiction Medicine, Kunming Medical University, Kunming 650500, China; 3 The First Department of Neurosurgery, The Sixth Affiliated Hospital, Kunming Medical University, People’s Hospital of Yuxi, Yunnan 653100, China; 4 Institute of Biological Science, Xi’ an Jiaotong-Liverpool University, Suzhou, Jiangsu, China; 5 Laboratory of Molecular Cardiology, Department of Cardiology, The First Affiliated Hospital of Kunming Medical University, Kunming 650032, China; 6 Department of Radiology, The Second Affiliated Hospital of Kunming Medical University, Kunming 650223, China

**Keywords:** glioma, BTN2A2, immune infiltration, cell proliferation, cell migration

## Abstract

Background: Protein casein 2A2 (BTN2A2) is a costimulatory molecule first identified in antigen-presenting cells. Studies have shown the involvement of BTN2A2 in immunity. However, the exact role and the mechanism of BTN2A2 in tumors are still unclear.

Methods: First, we performed real-time PCR to measure BTN2A2 expression in glioma cell lines. Next, we performed Genes Ontology (GO) and Kyoto Encyclopedia of Genes and Genomes (KEGG) enrichment analyses to understand the mechanism of BTN2A2 in glioma. Next, we used the “ESTIMATE”, “ssGSEA” and “CIBERSORT” algorithms to analyze the correlation between BTN2A2 and immune cell infiltration (ICI). Finally, we performed immunohistochemistry, growth curve, transwell, and colony formation assays to determine the functions of BTN2A2 in glioma.

Results: Our results showed an increase in BTN2A2 expression levels in glioma tissues and cells. Next, we determined that BTN2A2 was correlated with the prognosis of patients with glioma. Then, using the ESTIMATE, ssGSEA, and CIBERSORT algorithms, we discovered that BTN2A2 was significantly associated with immune cell infiltration (ICI) in glioma. We observed an increase in BTN2A2 expression levels with an increase in the patient’s tumor grade. Furthermore, BTN2A2 significantly enhanced the proliferative and migratory abilities of glioma cells.

Conclusions: Our results showed a significant increase in BTN2A2 expression levels in glioma cells and tissues. Furthermore, the prognosis of patients expressing high BTN2A2 levels was poor. Moreover, BTN2A2 was correlated with progression and ICI in patients with glioma. Together, this indicates that BTN2A2 could be a therapeutic target for patients with glioma.

## INTRODUCTION

In humans, gliomas are among the most common type of cancer of the central nervous system, of which glioblastoma (GBM) is the most lethal type of glioma. World Health Organization (WHO) grade IV gliomas account for 70% of all disseminated gliomas. However, the survival of patients with grade IV gliomas is 15 months [1]. Only two standard therapeutic approaches, including maximum safe surgical resection and combined chemoradiotherapy, are available for patients with glioma [2]. Temozolomide (TMZ) is the first-line chemotherapeutic drug widely used for treating patients with glioma. However, chemoresistance in patients with gliomas is primarily responsible for treatment failure. Highly active O_6_-methylguanine DNA methyltransferase (MGMT) could reduce the effect of alkylating agents, such as TMZ, thereby enhancing the tolerance of patients to chemotherapy. Furthermore, mutations in epidermal growth factor receptor, Galectin-1, mouse double minute 2, p53, and phosphatase and tensin homolog deleted on chromosome ten and changes in the activity of related signaling pathways are involved in regulating the progression of gliomas [3]. Significant advancements have been made in diagnosing or treating patients with glioma. Additionally, methylation in MGMT promoter [4], deletion in chromosome 1p and 19q [5], and isocitrate dehydrogenase (IDH) 1 and IDH2 mutations [6] are used for diagnosing or treating patients with glioma. Despite these advancements, the survival rate of patients has not significantly improved. Therefore, identifying new biomarkers or therapeutic targets for treating patients with glioma is an urgent need.

Butyrophilin is the major protein associated with fat droplets in milk [7]. Protein casein 2A2 (BTN2A2) is a costimulatory molecule. It was first discovered in antigen-presenting cells and involved in immunity [8]. Nevertheless, the role and the mechanisms of BTN2A2 in tumor onset and progression are yet to be determined.

In this study, we demonstrated that BTN2A2 expression was significantly high in glioma tissues and cell lines. BTN2A2 regulates glioma development via several immune-related signaling pathways. Furthermore, we used several algorithms and demonstrated a significant correlation between BTN2A2 and immune cell infiltration (ICI). Our study is the first to determine the involvement of BTN2A2 in glioma, thus suggesting that BTN2A2 could be a therapeutic target for treating patients in the future.

## MATERIALS AND METHODS

### Dataset retrieval and bioinformatics analysis

Transcriptomic and clinical information of 703 patients with glioma (LGG + GBM) were retrieved from the Cancer Genome Atlas (TCGA) database. Next, we performed Kaplan-Meier (KM) analysis to determine the patient’s overall survival (OS). We used the Tumor immune estimation resource (TIMER) (https://cistrome.shinyapps.io/timer/) [9], Gene expresssion profiling interactive analysis (GEPIA) (http://gepia.cancer-pku.cn/) [10], and the Chinese glioma genome atlas (CGGA) (http://www.cgga.org.cn/) databases to determine BTN2A2 expression in tumor and normal tissue. In addition, the retrieved GSE34152, GSE50161, and GSE66354 database (https://www.ncbi.nlm.nih.gov/gds) [11]. We evaluated the RNA seq data using the Xiantaoxueshu database (https://www.xiantao.love/writings). The significance of BTN2A2 in predicting patient prognosis was determined using the mRNAseq_325 obtained from CGGA and Rembrandt as well as Phillips datasets.

### Gene function enrichment analysis

The Gene Ontology (GO) and Kyoto Encyclopedia of Genes and Genomes (KEGG) pathway enrichment analysis to determine biological processes (BPs) and molecular functions (MFs) enriched by BTN2A2 among patients from TCGA. Gene set enrichment analysis (GSEA) was conducted to explore the functions and underlying mechanisms of BTN2A2 based on patients from the TCGA cohort.

### Correlation between BTN2A2 and tumor immune microenvironment (TIME) in glioma

To study the correlation between BTN2A2 and ICI, we analyzed the data on ICI in patients from the TCGA cohort using the “Cell-type Identification by Estimating Relative Subsets of RNA Transcripts” (CIBERSORT) algorithm.

### Cell cultures

We purchased glioma cells, including HAC, T98G, LN229, A172, and SF295 from the Kunming Institute of Zoology Cell Bank and cultured them in DMEM (Corning, NY, USA) supplemented with 1% penicillin/streptomycin and 10% fetal bovine serum at 37°C, 95% air, and 5% CO_2_.

### Cell transfection

Control and BTN2A2 siRNA plasmids were synthesized by GenePharma (Shanghai, China). We seeded SF295 and LN229 cells in to a 6-well plate and transfected them with siRNA using Lipofectamine 3000 reagent (Invitrogen, CA, USA). Scrambled siRNA was used as the negative control.

BTN2A2 siRNA sequence: GGAGAUGUUUGGAAACCAAUA.

### Quantitative real-time polymerase chain reaction (qRT-PCR)

We isolated total RNA using the TRIzol reagent (Invitrogen). Next, we reverse transcribed RNA using EasyScript First-Strand cDNA Synthesis SuperMix Kit (TransGen Biotech, China) and performed RT-qPCR with the aid of FastStart Universal SYBR Green Master Mix (Roche, TIANGEN Biotech, Beijing, China) on applied Biosystems 7500.

The sequences of primers are as follows: BTN2A2-FP: CTGTCATCCTGACCGCATCT, BTN2A2-RP: CGCAATTCTTCTTGAAGTTGCT, β-actin-FP: CTTCGCGGGCGACGAT, β-actin-RP: CCATAGGAATCCTTCTGACC.

Finally, we measured gene expression levels using the 2^−ΔΔCt^ method.

### Immunohistochemistry (IHC)

First, we dewaxed and treated the slides using 3% hydrogen peroxide. Next, we performed antigen retrieval using an antigen retrieval kit (Transgenitor bio) and blocked the proteins using 5% BSA (Bioss). Subsequently, the slides were incubated with the diluted primary antibody (anti-BTN2A2, ab233763, Abcam) overnight. Finally, the sections were stained with a DAB kit (K5007, Agilent Technologies, CA, USA) and counterstained using hematoxylin. The slides were visualized using a laboratory microscope (Nikon, Japan).

### Growth Curve

We seeded 1 × 10^4^ cells in 6-well plates for 24 hours and digested them with trypsin. Next, we extracted 20 μL cells, placed them on slides, and counted the cells daily for six days.

### Colony formation experiment

We seeded the cells transfected with siRNA in 6-well plates. We changed the medium every two days for 14 days. Next, we fixed the cells using a fixative, stained them with crystal violet, and photographed them for statistical analysis.

### Transwell assay

We used a transwell cell culture system to determine the migratory ability of cancer cells. First, we suspended 1 × 10^5^ cells in 1 mL serum-free DMEM. Next, we seeded 100 μL cells suspension on a transwell chamber and cultured it for 24 hours. Subsequently, we fixed the cells with 4% paraformaldehyde for 20 minutes, stained them using crystal violet, and counted the cells.

### Statistical analysis

We determined BTN2A2 expression in tumors using the TIMER, GEPIA 2, and TCGA databases. We performed the survival analysis using the log-rank test. Next, the correlation between genes was assessed using the *P*-value and Spearman correlation modulus. Furthermore, we determined BTN2A2 expression in normal glial and tumor cells using analysis of variance. *P* < 0.05 indicated the significance level.

### Data availability statement

GBMLGG (Glioma) data from the RNAseq data of level 3 HTSeq - FPKM format in TCGA (https://portal.gdc.cancer.gov/) (glioma) project. The gene expression profiling data sets (GSE34152), (GSE50161), and (GSE66354) were obtained from the GEO database (https://www.ncbi.nlm.nih.gov/gds), and they were used to analyze the differential expression of genes.

## RESULTS

### BTN2A2 expression in patients with cancer

**Figure 1:**
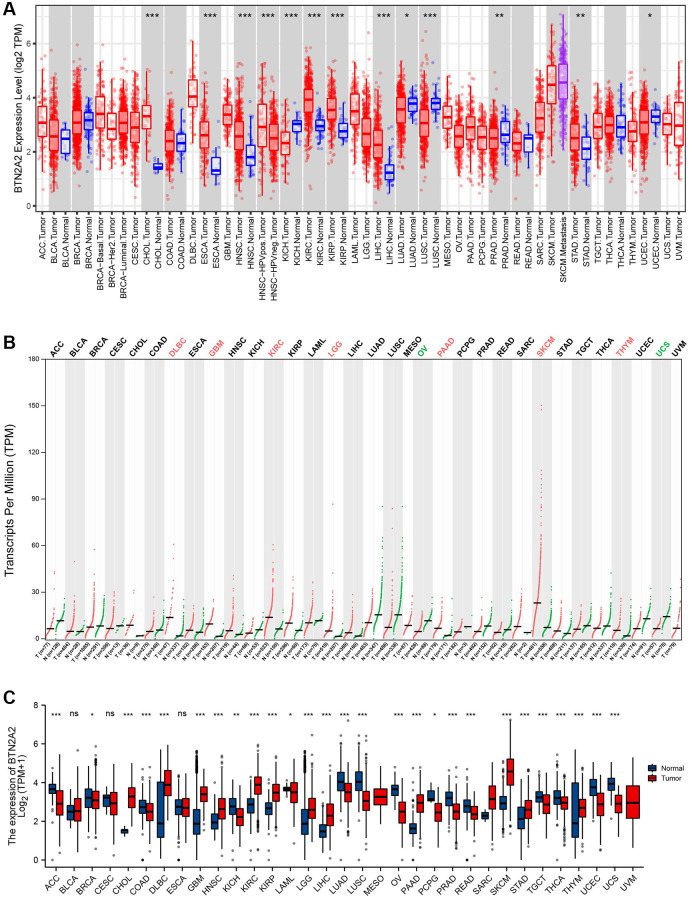
**BTN2A2 expression in patients with cancer.** (**A**) BTN2A2 expression in pan-cancer using TIMER. (**B**) BTN2A2 expression in pan-cancer using GEPIA 2. (**C**) BTN2A2 expression in pan-cancer using TCGA. Abbreviations: ACC: Adrenocortical carcinoma; BRCA: Bladder Urothelial carcinoma; CESC: Cervical squamous cell carcinoma and endocervical adenocarcinoma; DLBC: Lymphoid Neoplasm Diffuse Large B-cell Lymphoma; LAML: Acute Myeloid Leukemia; LUSC: Lung squamous cell carcinoma; MESO: Mesothelioma; PAAD: Pancreatic adenocarcinoma; PCPG: Pheochromocytoma and Paraganglioma; READ: Rectum adenocarcinoma; SARC: Sarcoma; TGCT: Testicular Germ Cell Tumors; THYM: Thymoma; UCS: Uterine carcinosarcoma; UVM: Uveal Melanoma; BLCA: Bladder urothelial carcinoma; CHOL: Cholangiocarcinoma; COAD: Colon adenocarcinoma; ESCA: Esophageal carcinoma; HNSC: Head and neck squamous cell carcinoma; KICH: Kidney Chromophobe; KIRP: Kidney renal clear cell carcinoma; KIRC: Kidney renal clear cell carcinoma; LIHC: Liver hepatocellular carcinoma; LUAD: Lung adenocarcinoma; PRAD: Prostate adenocarcinoma; SKCM: Skin Cutaneous Melanoma; STAD: Stomach adenocarcinoma; THCA: Thyroid carcinoma; UCEC: Uterine corpus endometrial carcinoma. ^*^P < 0.05, ^**^P < 0.01, ^***^P < 0.001.

We determined the difference in BTN2A2 expression in tumor and normal tissues using the differential gene expression module of TIMER. The results demonstrated an increase in BTN2A2 expression in patients with GBM, LGG, CHOL, HNSC, KIRC, LIHC, KIRP, STAD, DLBC, PAAD, SARC, SKCM and THYM. However, our results revealed a decrease in BTN2A2 expression in patients with KICH, LUAD, LUSC, PRAD, OV, UCS, UCEC, ACC, BRCA, COAD, PCPG, READ, TGCT, and THCA (Figure 1A–1C). Aberrant BTN2A2 expression in various cancers indicates that BTN2A2 could be an important cancer biomarker. However, the functions of BTN2A2 in glioma is still unclear.

### BTN2A2 expression in glioma and the correlation between BTN2A2 expression and clinicopathological features (CFs) of patients with glioma

**Figure 2:**
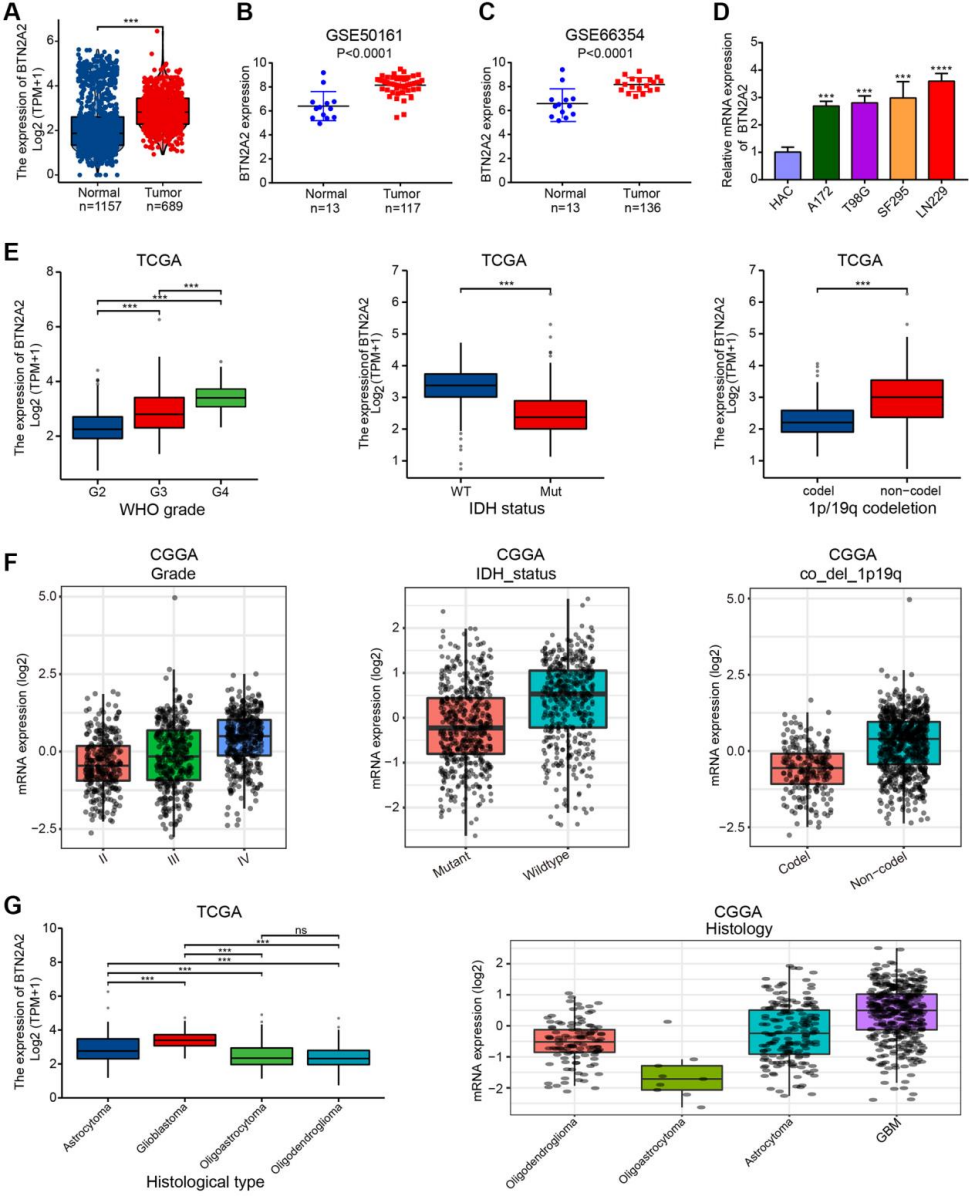
**Correlation between BTN2A2 expression and CFs.** (**A**) BTN2A2 expression in patients with glioma in TCGA cohort. (**B**, **C**) BTN2A2 expression in patients with glioma in GEO cohort. (**D**) BTN2A2 expression in normal human astrocytes cells (NHA) and glioma cells (A172, T98G, LN229, and SF295) was determined using qRT-PCR. (**E**–**G**) Correlation between BTN2A2 expression and CFs (grade, IDH status, 1p/19q codeletion, age, primary therapeutic outcome, and histological type). ^*^P < 0.05, ^**^P < 0.01, ^***^P < 0.001.

Our analysis showed a significant increase in BTN2A2 expression in patients with glioma from TCGA and GEO cohorts (Figure 2A–2C). Next, we performed qRT-PCR to determine BTN2A2 expression in glioma cell lines. We observed an increase in BTN2A2 expression in glioma cells (Figure 2D). Further, we observed a significant correlation between BTN2A2 and CFs, such as WHO grade, the status of IDH and 1p/19q codeletion in patients with glioma from TCGA and GEO cohorts. The results demonstrated a significant difference in BTN2A2 expression in patients with different tumor grades, tissue categories, and IDH mutations as well as 1p/19q codeletion status (Figure 2E–2G). Finally, logistics regression analysis and baseline data showed a significant correlation between BTN2A2 expression and CFs of patients in TCGA cohort (Tables 1 and 2). These results indicate the significance of BTN2A2 in clinical settings.

**Table 1:** Correlation between BTN2A2 and CFs of patients in TCGA cohort.

**Clinicopathological features**	**Low BTN2A2 expression**	**High BTN2A2 expression**	***P*-value**
*n*	348	348	
WHO grade, *n* (%)			
G2	175 (27.6%)	49 (7.7%)	<0.001
G3	118 (18.6%)	125 (19.7%)	
G4	13 (2%)	155 (24.4%)	
IDH status, *n* (%)			
WT	37 (5.4%)	209 (30.5%)	<0.001
Mut	308 (44.9%)	132 (19.2%)	
1p/19q codeletion, *n* (%)			
codel	144 (20.9%)	27 (3.9%)	<0.001
non-codel	203 (29.5%)	315 (45.7%)	
Primary therapy outcome, *n* (%)			
PD	52 (11.3%)	60 (13%)	<0.001
SD	96 (20.8%)	51 (11%)	
PR	42 (9.1%)	22 (4.8%)	
CR	101 (21.9%)	38 (8.2%)	
Age, *n* (%)			
≤60	312 (44.8%)	241 (34.6%)	<0.001
>60	36 (5.2%)	107 (15.4%)	
Histological type, *n* (%)			
Astrocytoma	96 (13.8%)	99 (14.2%)	<0.001
Glioblastoma	13 (1.9%)	155 (22.3%)	
Oligoastrocytoma	91 (13.1%)	43 (6.2%)	
Oligodendroglioma	148 (21.3%)	51 (7.3%)	
Age, median (IQR)	40 (32, 51)	53 (37, 63)	

**Table 2:** Correlation between BTN2A2 and CFs of patients.

**Clinicopathological features**	**Total (*N*)**	**Odds ratio (OR)**	***P*-value**
WHO grade (G3&G4 vs. G2)	635	7.634 (5.263–11.236)	<0.001
IDH status (Mut vs. WT)	686	0.076 (0.050–0.113)	<0.001
1p/19q codeletion (non-codel vs. codel)	689	8.276 (5.371–13.180)	<0.001
Primary therapy outcome (SD vs. PD)	259	0.460 (0.277–0.759)	0.003
Age (>60 vs. ≤60)	696	3.848 (2.568–5.882)	<0.001
Histological type (Glioblastoma vs. Astrocytoma)	363	11.562 (6.348–22.658)	<0.001

### Significance of BTN2A2 in predicting the prognosis of patients with glioma

**Figure 3:**
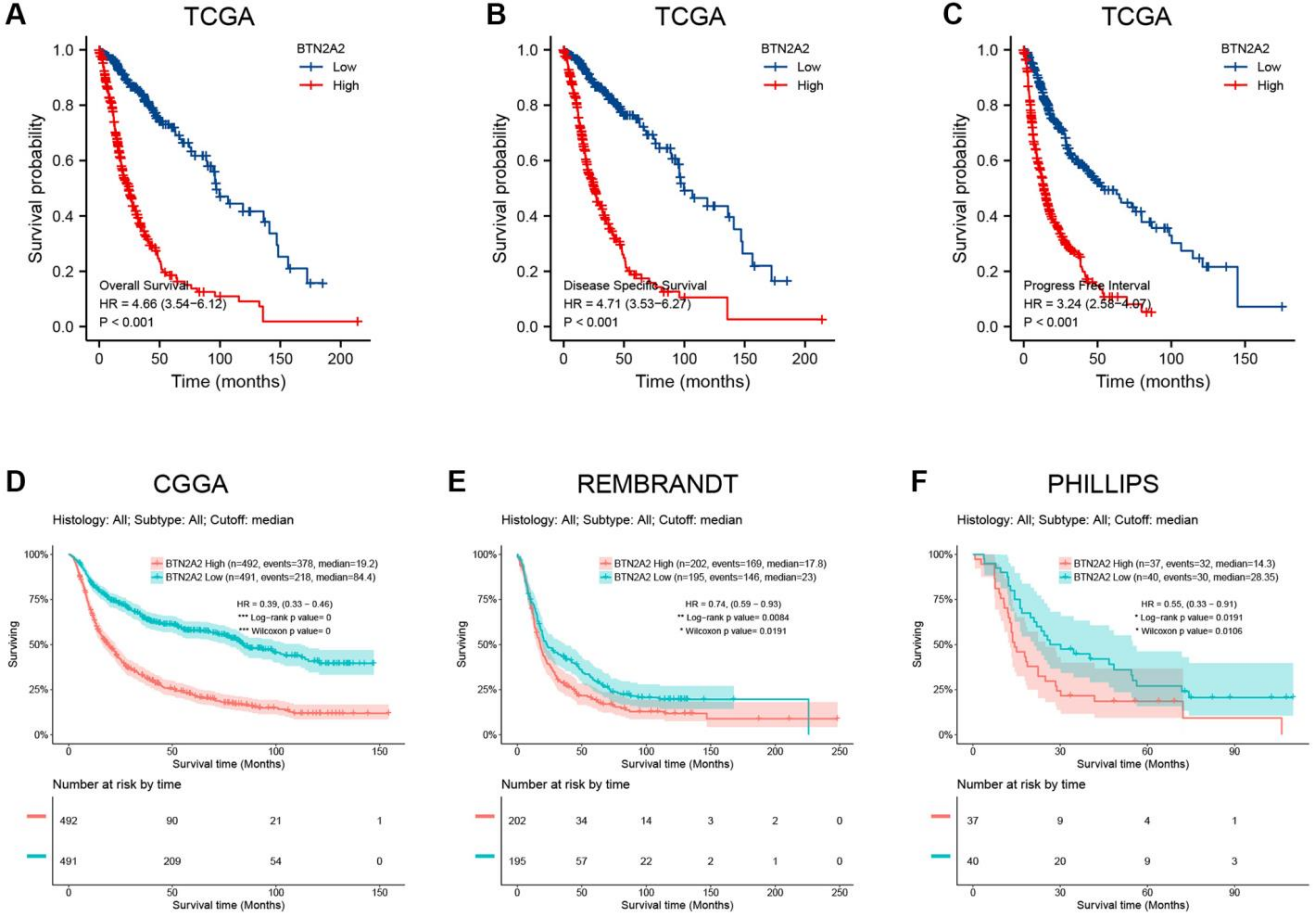
**Survival curve for determining the prognostic significance of BTN2A2.** (**A**–**C**) The significance of BTN2A2 in predicting the prognosis of patients with glioma from the TCGA cohort. (**D**–**F**) The significance of BTN2A2 in predicting the prognosis of patients with glioma using CGGA, REMBRANDT, and Phillips databases.

**Figure 4:**
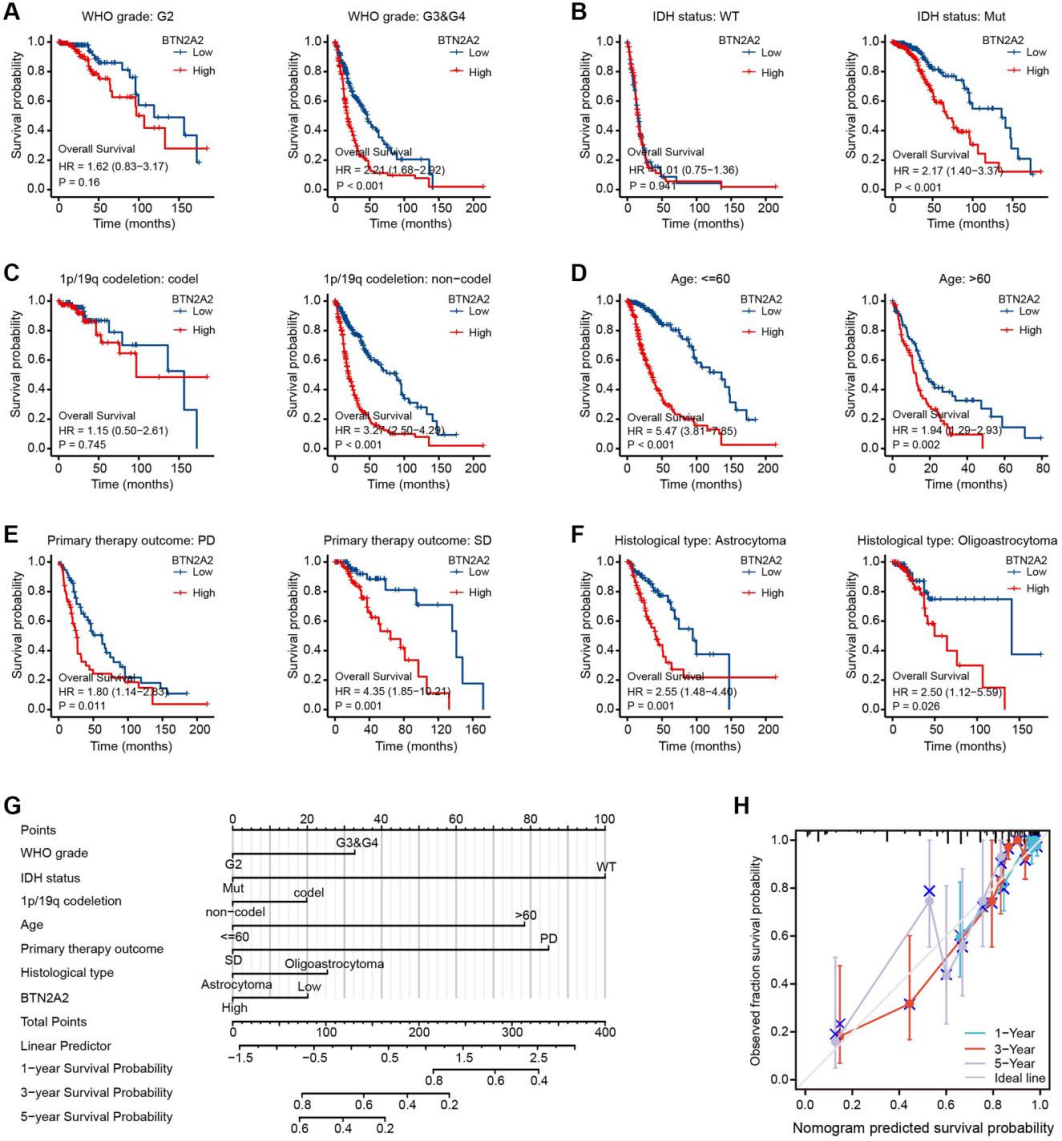
**Analyzing the prognostic value of BTN2A2 in different glioma subgroups.** (**A**–**F**) Correlation between total survival and glioma grade, IDH mutation, and 1p/19q codeletion status, age, primary therapeutic outcome, and histological types. ^*^P < 0.05, ^**^P < 0.01, ^***^P < 0.001. (**G**) Construction of a nomogram to determine the OS of patients with glioma. (**H**) The calibration curve was used to determine the OS of patients from the TCGA cohort.

Next, we determined the significance of BTN2A2 in predicting the prognosis of patients with glioma. Hence, we determined the effect of BTN2A2 expression on the survival of patients using TCGA, CGGA, Rembrandt, and Phillips datasets. The results demonstrated that the survival of patients expressing low BTN2A2 levels was good and the survival of patients expressing high BTN2A2 levels was poor (Figure 3A–3F). We used the KM (Figure 4A–4F), Nomogram (Figure 4G), and Calibration analysis (Figure 4H) to investigate the significance of BTN2A2 in predicting the prognosis of patients with different CFs. A significant correlation between the effect of BTN2A2 on the patient prognosis and age, the status of IDH mutation, 1p/19q co-deletion, tumor grade, primary therapy outcome, and type of tumor tissues. These results indicate that BTN2A2 could be used for predicting the patient’s prognosis.

### GSEA of BTN2A2 in patients with glioma

**Figure 5:**
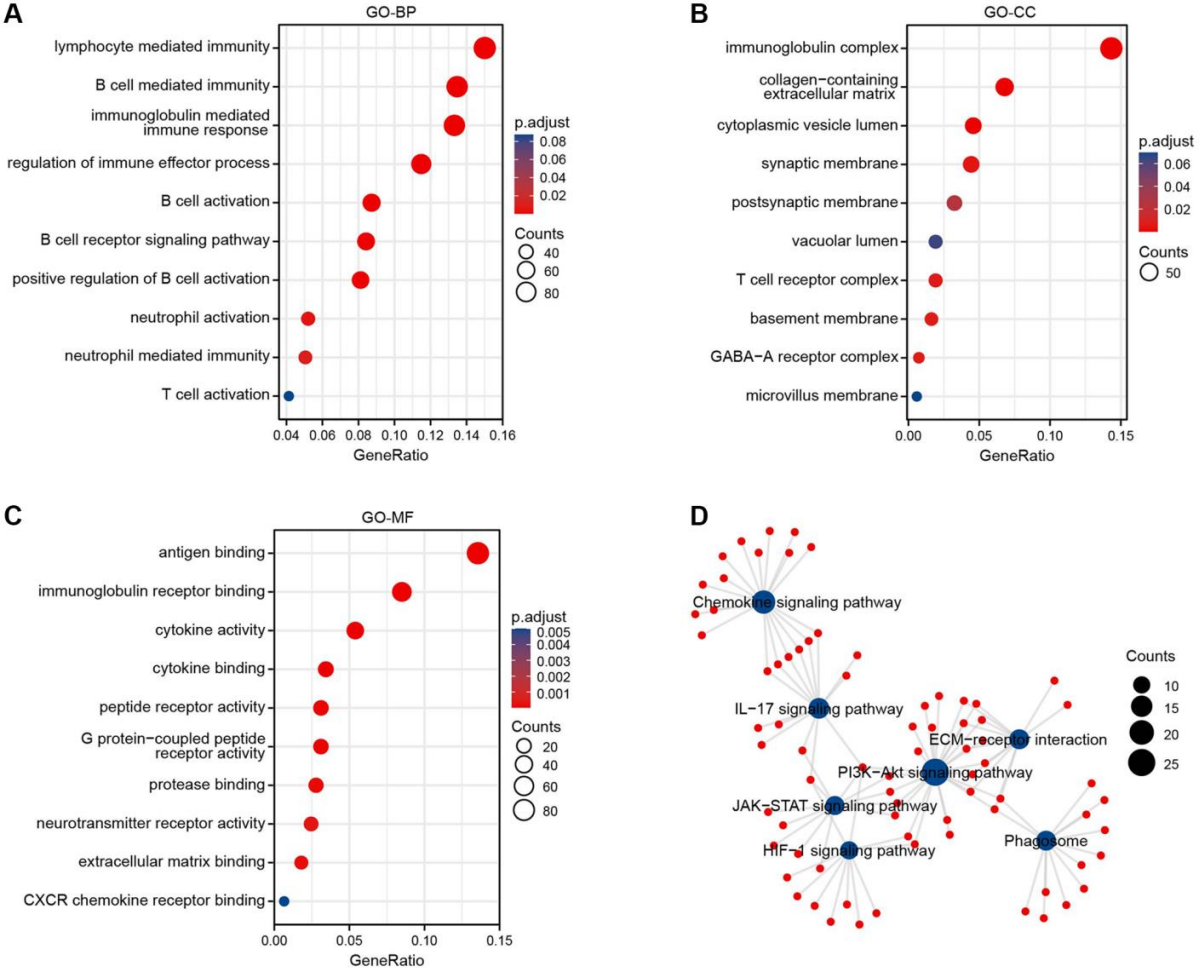
**Enrichment analysis of BTN2A2 in glioma.** (**A**–**C**) The GO enrichment analysis was performed on BTN2A2 using TCGA. (**D**) The KEGG pathway analysis of BTN2A2 using the TCGA database.

We performed the GO and KEGG pathway enrichment analysis to determine the functions of BTN2A2. The GO-BP terms such as lymphocyte, B cells, and neutrophil-mediated immunity, immune responses mediated by immunoglobulin, immune effector process regulation, the activation of B cells, the B cell receptor signaling pathway, and the positive regulation of B cell activation were enriched by BTN2A2 (Figure 5A). The GO-cellular component (CC) terms enriched by BTN2A2 were immunoglobulin and T cell receptor complexes, collagen-containing extracellular matrix (ECM), cytoplasmic vesicles and vacuolar lumen, synaptic and postsynaptic membranes, and basement membrane, etc., (Figure 5B). The GO-MF terms, such as antigen binding functions, immunoglobulin receptor, protease, ECM, and the cytokine, peptide receptor, G protein-coupled peptide receptor (GPCR), and neurotransmitter receptor activities were enriched by BTN2A2 (Figure 5C).

The KEGG pathway enrichment analysis showed the enrichment of the chemokine, interleukin 17 (IL-17), phosphatidylinositide 3-kinases (PI3K-Akt), Janus kinase signal transduction and transcriptional activator (JAK-STAT), HIF-1 signaling pathways, the interaction between ECM-receptors, and phagosome were enriched by BTN2A2 (Figure 5D). Further, most of these pathways regulated immune responses. The JAK-STAT signaling pathway mediates immunomodulatory processes, including the recognition of tumor cells and tumor cells escaping immune surveillance [12]. The PI3K-AKT signaling pathway could attenuate the effector functions of immune cells and alter the tumor microenvironment (TME) [13]. A study showed highly active IL-17 signaling pathway was correlated to high CD8+ T cell infiltration and changes in the TIME in breast cancers [14].

**Figure 6:**
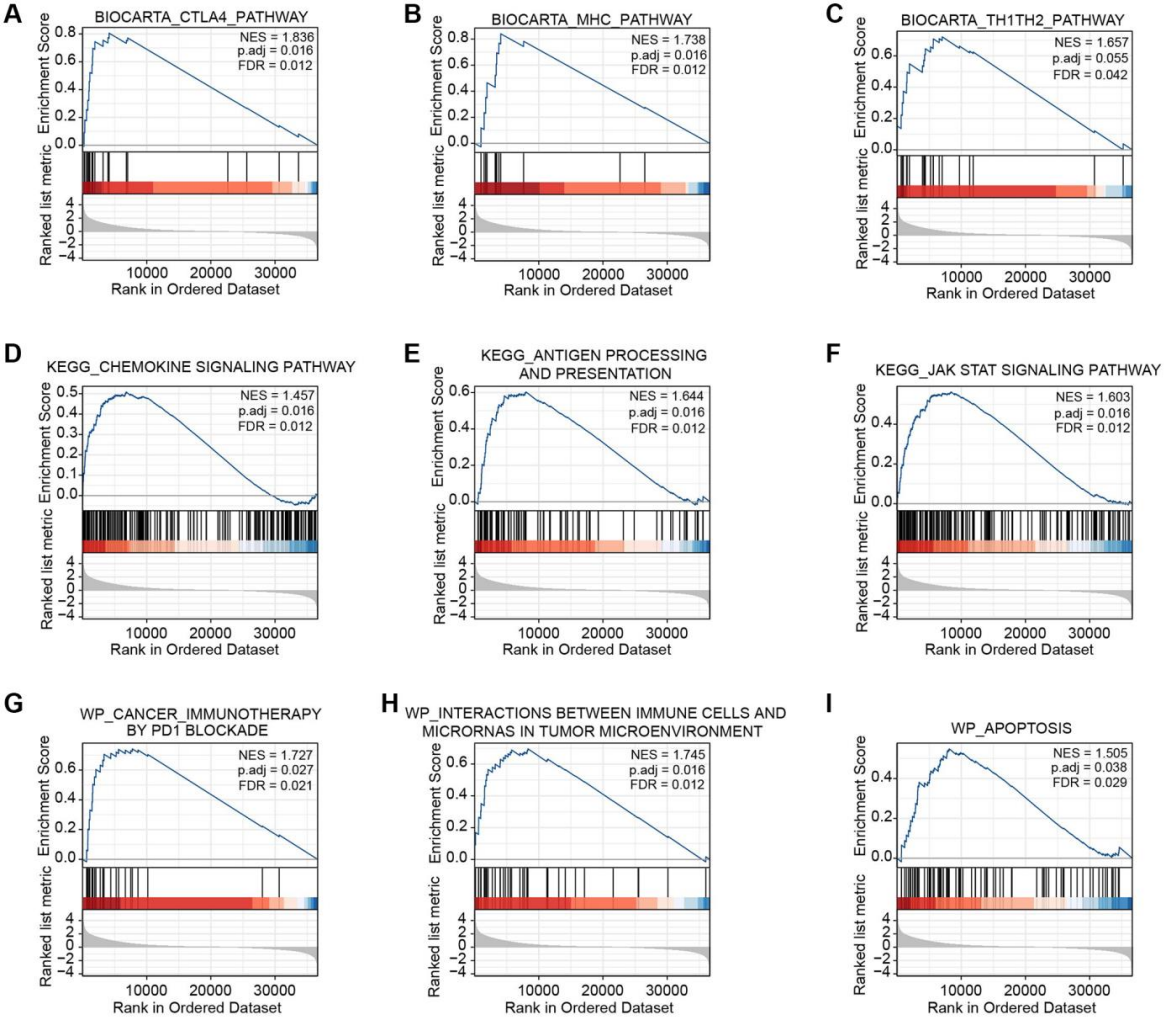
**GSEA of BTN2A2 in glioma.** (**A**–**I**) The CTLA4, MHC, Th1-Th2, chemokine, and JAK-STAT signaling pathway, the processing and presentation of antigens, and apoptosis were determined using GSEA.

We performed GSEA to determine the underlying mechanisms of BTN2A2 in patients with glioma in our cohort. The results showed BTN2A2 was associated with the CTLA4, MHC, chemokine, JAK-STAT signaling pathways, antigen processing and presentation, PD1 blockade cancer immunotherapy, immune cells-microRNAs interactions in the TME, and apoptosis (Figure 6A–6I).

These results showed a significant effect of BTN2A2 on activating immune system in patients with glioma.

### Role of BTN2A2 in the TIME

**Figure 7:**
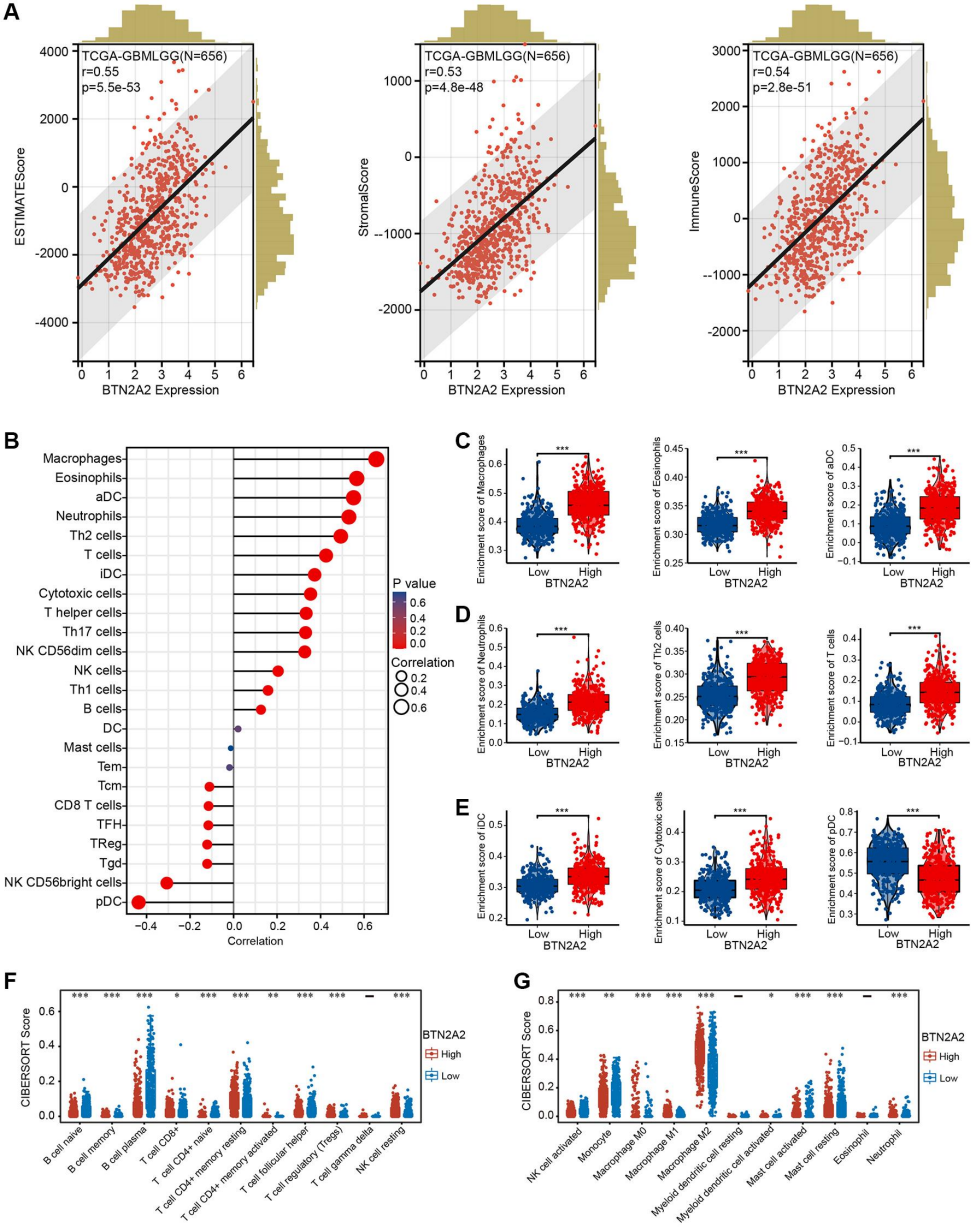
**Correlation between BTN2A2 and ICI in patients with glioma.** (**A**) Correlation between BTN2A2 and stromal, immune, and ESTIMATE scores of patients with glioma using ESTIMATE algorithm. (**B**–**E**) Correlation between BTN2A2 and ICIs in patients with glioma using the ssGSEA algorithm. (**F**, **G**) Correlation between BTN2A2 and ICI in patients with glioma using the CIBERSORT algorithm. ^*^P < 0.05, ^**^P < 0.01, ^***^P < 0.001.

The GO and KEGG pathway enrichment analysis demonstrated correlation between BTN2A2 and immune regulation. Hence, we determine the correlation between BTN2A2 expression and the ICI. The ESTIMATE results showed a high correlation between BTN2A2 expression and stromal cells as well as immune cells (Figure 7A). Next, we used the ssGSEA and CIBERSORT algorithms to determine the correlation between BTN2A2 expression and ICI. The results demonstrated a significant correlation between BTN2A2 and macrophages, activated, immature, myeloid, and plasmacytoid dendritic cells, neutrophils, CD8+ and CD4+ T cells, monocytes, T cell regulatory, type 1, 2, and 17 T helper (Th) cells, B cells, cytotoxic cells, Th cells, natural killer cells, and follicular Th cells (Figure 7B–7G).

**Figure 8:**
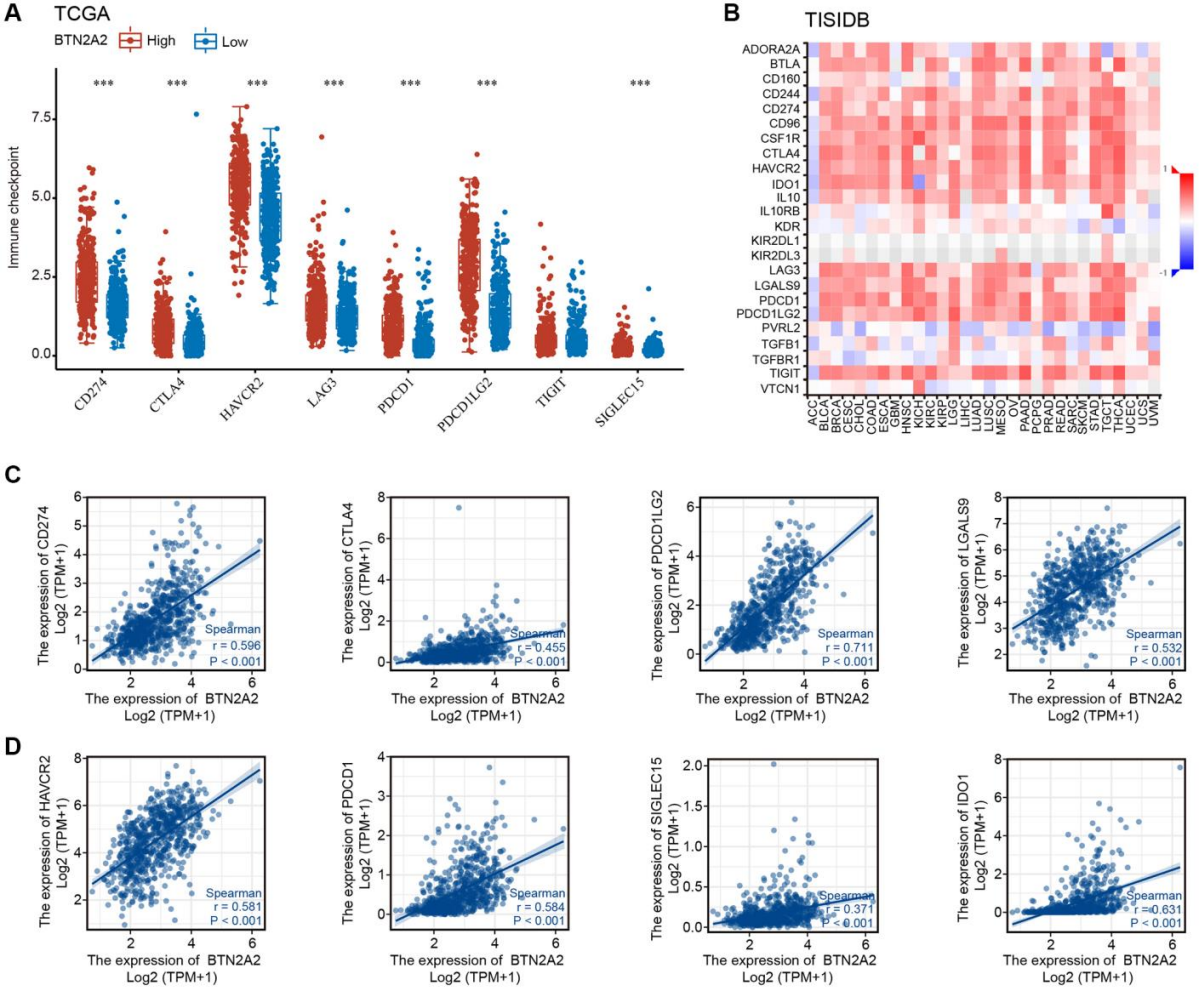
**The correlation between BTN2A2 expression and immune checkpoint genes.** (**A**, **B**) Correlation between BTN2A2 expression and immune checkpoint genes in patients with glioma using TCGA and TISIDB databases. (**C**, **D**) Analyzing the correlation between BTN2A2 expression and immune checkpoint genes in patients with glioma using Spearman^’^s correlation.

Immune checkpoint blocking (ICB) is used as a therapeutic strategy for treating patients with cancers [12]. ICB therapy has revolutionized cancer treatment [13]. Therefore, we investigated the correlation between BTN2A2 expression and immune checkpoint molecules, such as PDCD1LG2 (PD-L2), CD274 (PD-L1), PDCD1 (PD-1), TIGIT, SIGLEC15, IDO1, LAG3, CTLA-4, and HAVCR2. The results showed a significant positive correlation between BTN2A2 and immune checkpoint gene expression (Figure 8A–8D), thereby indicating the significance of BTN2A2 in the immunotherapy of glioma.

### Knockdown of BTN2A2 inhibits the migration and proliferation of glioma cells

**Figure 9:**
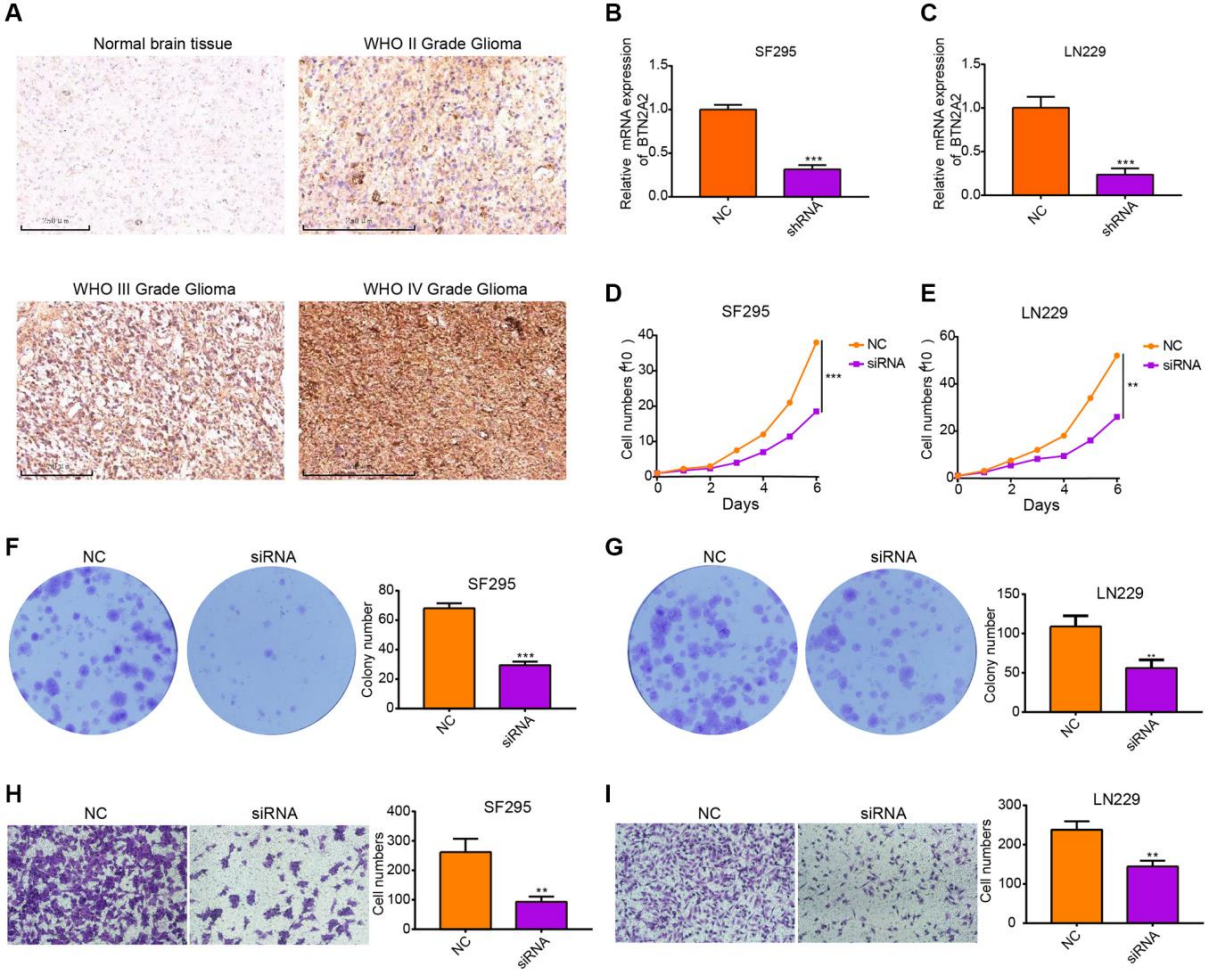
**Knockdown of BTN2A2 expression inhibits the proliferation and migration of glioma cells.** (**A**) IHC shows BTN2A2 expression in glioma and normal brain tissues. (**B**, **C**) The efficacy of BTN2A2 knockdown in SF295 and LN229 using qRT-PCR. (**D**, **E**) BTN2A2 knockdown inhibits the growth of SF295 and LN229 cells determined using growth curve. (**F**, **G**) BTN2A2 knockdown inhibits the migration of SF295 and LN229 cells determined using the colony information assay. (**H**, **I**) BTN2A2 knockdown inhibits the migration of SF295 and LN229 cells determined using the transwell assay. Scale bar = 250 um. Abbreviations: NC: Negative control; siRNA: Knockdown of BTN2A2. ^*^P < 0.05, ^**^P < 0.01, ^***^P < 0.001.

We performed IHC on three gliomas and one normal tissue. The results demonstrated an increase in BTN2A2 expression was observed with increasing glioma grade (Figure 9A). To determine the functions of BTN2A2 in glioma cells, BTN2A2 expression was knockdown in SF295 and LN229 cells using siRNA. Next, we performed qRT-PCR to determine knockdown efficiency (Figure 9B, 9C). The growth curve and colony formation assays showed that the knockdown of BTN2A2 could inhibit SF295 and LN229 cell proliferation (Figure 9D–9G). Next, we performed a transwell assay to determine if BTN2A2 could regulate the migration of cancer cells. The results demonstrated a significant decrease in the migratory abilities of BTN2A2 knockdown cells compared to control cells (Figure 9H, 9I). Together, this indicates that BTN2A2 overexpression could promote the proliferative and migratory abilities of glioma cells.

## DISCUSSION

In gliomas, the status of IDH mutations and 1p/19q co-deletion are widely used markers based on WHO guidelines (2016) for predicting the patient prognosis [14]. In addition, the mutation in TERT promoters is considered a molecular hallmark of GBM and is frequently used as a biomarker for predicting the prognosis of patients with GBM [15]. Patients with WHO grade II and III gliomas and secondary GBM (WHO Grade IV) frequently harbor IDH mutations; However, the IDH mutation rate in patients with primary GBM (WHO Grade IV) is low [6, 16–18]. The deletion in chromosomal 1p/19q is primarily detected in patients with oligodendrogliomas; However, chromosomal 1p/19q deletion is rare in patients with astrocytomas [19, 20]. Therefore, identifying new markers for predicting the patient prognosis to improve the diagnosis and treatment outcomes is the need of the hour.

We used bioinformatics analysis to determine the involvement of BTN2A2 in patients with glioma, including CFs, prognosis, and functional analysis. We observed an increase in BTN2A2 expression in patients from GEO and TCGA cohorts. Furthermore, IHC analysis demonstrated an increase in BTN2A2 expression in glioma tissues compared to normal brain tissues. In addition, the OS of patients expressing high BTN2A2 levels was poor compared to patients expressing low BTN2A2 levels. The GO-BP terms enriched by BTN2A2 were immunity mediated by lymphocytes, neutrophils, and B cells, immune responses mediated by immunoglobulin, the regulation of immune effector process, the activation of B cells, the B cell receptor signaling pathway, and the positive regulation of B cell activation. The GO-CC terms, including immunoglobulin complex, collagen-containing ECM, cytoplasmic vesicle and vacuolar lumen, synaptic and postsynaptic membranes, T cell receptor complex, and basement membrane, etc., were enriched by BTN2A2. Furthermore, the GO-MF terms include antigen binding functions, immunoglobulin receptor, cytokine, protease, and ECM binding, the cytokine, peptide receptor, GPCR, and neurotransmitter receptor activities were enriched by BTN2A2. The KEGG pathway enrichment analysis showed the enrichment of the chemokine, IL-17, JAK-STAT, PI3K-Akt, and HIF-1 signaling pathway, the interaction between ECM-receptor and phagosome. Next, we performed GSEA on our cohort to determine the underlying mechanisms of BTN2A2 in glioma. The results revealed BTN2A2 was associated with the CTLA4, MHC, chemokine, and JAK-STAT signaling pathway, the processing and presentation of antigens, PD1 blockade immunotherapy, interactions between immune cells and microRNAs in the TME, and apoptosis.

Recently, immunotherapy has emerged as a new and attractive platform for treating patients with glioma [21]. However, immunotherapy for treating patients with glioma in clinical settings is ineffective [22]. Therefore, identifying a new marker as a target for immunotherapy for patients with glioma is the need of the hour. Hence, we used the ESTIMATE, ssGSEA, and CIBERSORT algorithms to determine the correlation between BTN2A2 and immune cells. Our results showed a high correlation between BTN2A2 and ICIs in patients with gliomas. In the late 19th century, the concept of immunotherapy was proposed, which refers to using an individual’s immune system to attack and destroy cancer cells [13]. ICB therapy has revolutionized cancer treatment [23]. A study has showed that immune checkpoint gene expression restricts immune surveillance in the TME [24]. Studies have shown that antibodies against CD274, CTLA-4, and PDCD1 (immune checkpoint molecules) could promote antitumor T cell activity and improve clinical outcomes in various cancers [25–28]. Our results showed an association between BTN2A2 and CTLA-4, CD274, HAVCR2, LAG3, PDCD1LG2, PDCD1, and SIGLEC15 (immune checkpoint molecules) in patients with glioma. These results indicate that BTN2A2 could be a potential immunomarker for patients with glioma. Importantly, our results demonstrated that knockdown of BTN2A2 could significantly inhibit the proliferative and migratory abilities of glioma cells. Together, our results reveal the significance of BTN2A2 in glioma. This indicates that BTN2A2 could be used as a marker for predicting prognosis and serve as a novel therapeutic target in the future.

## CONCLUSION

In conclusion, our results showed an increase in BTN2A2 expression in glioma cells and tissues. Further, the prognosis of patients expressing high BTN2A2 levels was poor. Importantly, we showed a correlation between BTN2A2, progression, and ICI in patients with glioma. Together, our results suggest that BTN2A2 could be a therapeutic target for patients with glioma.
